# Misuse and diversion of stimulant medications prescribed for the treatment of ADHD: a systematic review

**DOI:** 10.3389/fpsyt.2025.1612785

**Published:** 2025-06-30

**Authors:** Jessica Forrest, Winnie Chen, Karuppiah Jagadheesan

**Affiliations:** ^1^ Wyndham Clinic, Mercy Mental Health, Melbourne, VIC, Australia; ^2^ Leeder Centre for Health Policy Economics and Data, Faculty of Medicine and Health, University of Sydney, Sydney, NSW, Australia; ^3^ Menzies School of Health Research, Charles Darwin University, Darwin, NT, Australia; ^4^ NorthWest Adult Mental Health Service, Northern Health, Melbourne, VIC, Australia; ^5^ Department of Human Psychopharmacology, Swinburne University, Melbourne, VIC, Australia

**Keywords:** ADHD, diversion, psychotropic medications, stimulant misuse, systematic review

## Abstract

**Aim:**

Stimulant medications are an evidence-based treatment for ADHD. However, stimulant medications are associated with a risk of misuse and diversion. Given the paucity of research, this systematic review evaluates the prevalence of misuse and diversion of stimulant medication by those who are prescribed the medication for a diagnosis of ADHD, and explores risk factors for misuse and diversion.

**Methods:**

This systematic review was registered with PROSPERO (CRD42023469041). A systematic search of original articles from PubMed and PsycInfo on the topic of interest over a period of 10 years (2012-2023) was conducted. Risk of bias was assessed through JBI Critical Appraisal Checklist for Prevalence Studies. A meta-analysis was conducted using JBI SUMARI software.

**Results:**

Twelve cross sectional surveys met the inclusion criteria, with study populations ranging from 88 respondents to 10,000 respondents. Meta-analysis found an average prevalence of past year prescription stimulant medication misuse of 22.6%, past year diversion of 18.2%, and lifetime diversion of 17.9%. Risk factors for misuse included being prescribed an amphetamine-based stimulant, reporting comorbid depressive and anxiety symptoms, and believing that misuse was not associated with risks. Risk factors for diversion included exposure to peers who were misusing stimulant medication, and having surplus medication available.

**Conclusion:**

With more than one in five people reporting misuse of their prescribed stimulant medication, and one in six diverting their prescribed stimulant medication, comprehensive risk assessment and risk mitigation strategies are needed. Further research in a variety of geographic and demographic settings is needed to develop effective risk assessment tools and targeted interventions.

## 1 Introduction

Attention-deficit/hyperactivity disorder (ADHD) is a neurodevelopmental disorder with onset in childhood. The 5th edition of the Diagnostic and Statistical Manual of Mental Disorders (DSM) defines the condition as a pattern of inattention and/or hyperactivity-impulsivity that interferes with functioning or development (1). Global prevalence of ADHD has been estimated at 7.2% in children and adolescents (2) and 6.76% in adults (3). In Australia, ADHD is the most common disorder in children with an estimated prevalence of 8.2% (4).

Stimulant medications, including methylphenidate and amphetamine-based medications, such as lisdexamfetamine, dexamfetamine, and dextroamfetamine/amfetamine, are recommended as the first line pharmacological treatment for ADHD (5, 6). Untreated ADHD is associated with life-long impairments in functioning, including poorer academic outcomes and educational achievement, increased road traffic accidents and other accidental injuries, greater challenges with parenting, and increased risk of substance use disorders (7, 8). Effective pharmacological treatment mitigates the risk of functional impairment associated with ADHD. There is evidence that stimulant treatment reduces suicide attempts in young people with ADHD and improves driving performance (8). Treating ADHD may reduce the risk of the person developing a substance use disorder, reduce the severity of a comorbid substance use disorder, and support recovery from substance use disorder (8). Despite clear benefits of stimulant treatment, and evidence and guidelines recommending stimulant treatment as first line therapy, treating practitioners report reluctance to prescribe stimulant medications due to concerns about misuse and diversion (9).

Stimulant medications are considered Schedule 8 controlled drugs in Australia, along with opioids, ketamine, alprazolam and flunitrazepam (10). Schedule 8 drugs ‘require restriction of manufacture, supply, distribution, possession and use to reduce abuse, misuse and physical or psychological dependence (11). The potential for misuse associated with stimulant medications relates to its pharmacological similarities to cocaine. Stimulant medications are capable of causing intoxication similar to cocaine (12) and a similar ‘high’ is described by patients who take stimulant medications intranasally and via injection. Whilst obtaining a ‘high’ is a common reason for misuse of stimulant medication, other commonly cited reasons include to address untreated symptoms of ADHD, to improve concentration and alertness, and for academic reasons (13, 14). Stimulant medications may also be misused for appetite suppression and weight loss (15).

Reasons for misuse among individuals prescribed stimulants for ADHD differ from those without a diagnosis of ADHD. For those with ADHD, enhancing alertness and improving academic or work performance may be more common drivers of misuse than recreational purposes (16). Individuals report diverting prescription stimulant medication for altruistic reasons (e.g. to help a friend or family member who has run out of their own medication or is in a time of academic stress) and for monetary gain (17). There are potential health risks associated with prescription stimulant use, and these risks are amplified when stimulants are misused or diverted. Stimulants activate the sympathetic nervous system and increase heart rate and blood pressure (11). Cardiovascular effects appear to be minimal in healthy populations, however, may be potentially serious in the presence of pre-existing cardiovascular conditions (18). Adverse psychiatric effects include mood changes, tics, anxiety, insomnia, and possible increased suicide risk (19). Misuse of stimulant medication has also been associated with cases of death as a result of individuals injecting medication (20, 21). Injection of any drug intended to be orally administered can be lethal due to vascular damage caused by insoluble substances (22). A series of deaths in Tasmania led the coroner to recommend, in a 2014 report, that ‘medical practitioners prescribing psychostimulant medication such as Ritalin continue to be vigilant in assessing the serious risks associated with such prescription’ and ‘that relevant agencies consider whether there is a need for a public education campaign with a view to reducing the harm caused by illicit diversion of psychostimulants, and in particular, to highlight the dangerous practice of intravenous injection of such substances’ (20).

Australian and overseas guidelines advise risk assessment for substance misuse and drug diversion before and during treatment for ADHD (5, 6). Guidelines direct practitioners to consider that stimulants may be diverted for cognitive enhancement or appetite suppression, and that immediate-release stimulants are more commonly diverted (6). However, the guidelines do not provide prevalence rates of misuse or diversion of stimulant medication among those prescribed stimulant medication for ADHD, and no specific risk assessment tool is recommended to guide the assessment.

Previous systematic reviews (13, 14, 23) have not focused on the subpopulation of people who are prescribed stimulant medication with a diagnosis of ADHD, and meta-analysis has not been performed. This systematic review aims to evaluate the prevalence of misuse and diversion of stimulant medication by those who are prescribed the medication for a diagnosis of ADHD and to explore risk factors for misuse and diversion within this population. The broad range of results across three systematic reviews, which were not focused on the population of interest, indicates that a focused systematic review with meta-analysis of prevalence rates may fill a gap in the literature. Improved understanding of the extent of misuse and diversion of stimulant medication by those prescribed the medication, and risk factors, may inform approaches and strategies aimed at mitigating these risks.

## 2 Methods

This review followed the Preferred Reporting Items for Systematic Reviews and Meta-Analyses (PRISMA) guidelines (24). The review protocol was registered on PROSPERO (CRD42023469041). The review aimed to address the following research questions:

1. Among individuals of all ages who are prescribed stimulant medication for treatment of ADHD, how many misuse or divert their medication?
2. Among individuals of all ages who are prescribed stimulant medication for treatment of ADHD, and who misuse or divert their medication, what risk factors differentiate these individuals from others who do not misuse or divert their medication?

For the purposes of this review, ‘misuse’ refers to the use of prescribed stimulants in excess of the prescribed dose, non-orally (intranasally or via injection), using for a purpose other than treatment of ADHD, or using with alcohol or other drugs. Other terms used in the literature include ‘abuse’, ‘non-medical use’ and ‘medical misuse’. ‘Diversion’ refers to the transfer of stimulants prescribed for ADHD from one individual who does have a prescription, to another who does not, typically by selling or giving away.

### 2.1 Eligibility criteria

Previous literature reviews on this topic have included articles from 1995 – 2006 (13), 1948 – 2011 (23) and 1948 – 2018 (14). However, none focused on the population of those who are prescribed stimulant medication for ADHD, nor performed a meta-analysis on prevalence rates. This systematic review included articles from 2012-2023. This search period was chosen to encompass 10 years of recent research in this area, noting that two previous reviews encompassed literature up to 2011 (14, 23).

Searches were limited to human studies in English and original research. Eligibility criteria were defined as follows: individuals of any age or setting who were currently or previously prescribed stimulant medication for a diagnosis of ADHD. Where ADHD diagnosis was not specified, if prescription status was assessed and the study otherwise met inclusion criteria, the study was included. This decision was taken due to the limited research in this area specifically recording ADHD diagnosis. Studies were eligible if they provided prevalence of misuse and diversion within those prescribed stimulant medication for ADHD, or sufficient data for this to be calculated.

The PECO (Population, Exposure, Comparator, Outcome) framework was used to define eligibility and guide data extraction:

- **Population**: Individuals prescribed stimulant medication for a diagnosis of ADHD.
- **Exposure**: Stimulant medication.
- **Comparator**: Those with a prescription for stimulant medication reporting misuse or diversion, compared to those not reporting misuse or diversion.
- **Outcome**: Prevalence of stimulant medication misuse and diversion, and associated risk factors.

### 2.2 Search strategy

The following search was utilized in PubMed, and translated for PsycInfo:

(misuse OR abuse OR nonmedical use OR inappropriate use OR illicit use OR diversion) AND (stimulant medications OR amphetamines OR methylphenidate) AND (ADHD OR attention-deficit hyperactivity disorder OR attention deficit disorder) AND (“2012/01/01”[Date - Publication]: “2023/07/01”[Date - Publication]).

### 2.3 Selection process and data extraction

Articles identified through the search strategy were uploaded into Covidence software (25) for screening and study selection. Titles and abstracts were screened independently by two authors (JF, WC), followed by full text screening of selected studies by both authors. Where a consensus was not reached, conflicts were resolved by the third author. Reasons for exclusion at full text stage were recorded. Where studies that appeared to meet inclusion criteria had data of interest that were missing, conflicting, or unclear, authors were contacted for clarification. If a response was not received within 2 weeks, the study was excluded.

Relevant data were extracted by one investigator using a template in Excel. The outcomes of interest were prevalence of misuse of prescription stimulant medication by those prescribed the medication for ADHD, prevalence of diversion of prescription stimulant medication by those prescribed the medication for ADHD, and risk factors for both. Studies were selected if they reported on one or more of these outcomes. Other data obtained included sample size and setting, study design, demographics of participants, terminology used to define the outcomes of interest, whether and how participants were assessed for ADHD, the total number of participants prescribed stimulant medication, and the number of these individuals who reported misuse or diversion. Risk factors for misuse and diversion by those with a prescription were also sought.

### 2.4 Risk of bias assessment

The 12 studies selected for inclusion were critically appraised using the JBI Critical Appraisal Checklist for Prevalence Studies (26). Two reviewers independently assessed methodology and disagreements were resolved through consensus (JF, WC).

### 2.5 Synthesis methods

Meta-analysis was performed utilizing prevalence rates of misuse and diversion from individual studies. JBI SUMARI software (27) was used to produce forest plots for visual representation of individual study results, to determine 95% confidence intervals of prevalence rates, and to perform statistical analysis of heterogeneity. Heterogeneity was quantified using the I2 statistic, with values over 50% indicating high heterogeneity. Risk factors for diversion and misuse of medication by those prescribed the medication were synthesized narratively. Meta-analysis was not performed for risk factors due to limited data and high heterogeneity in the studies. Studies with incomplete or missing data regarding the outcomes of interest were excluded.

**Figure 1:**
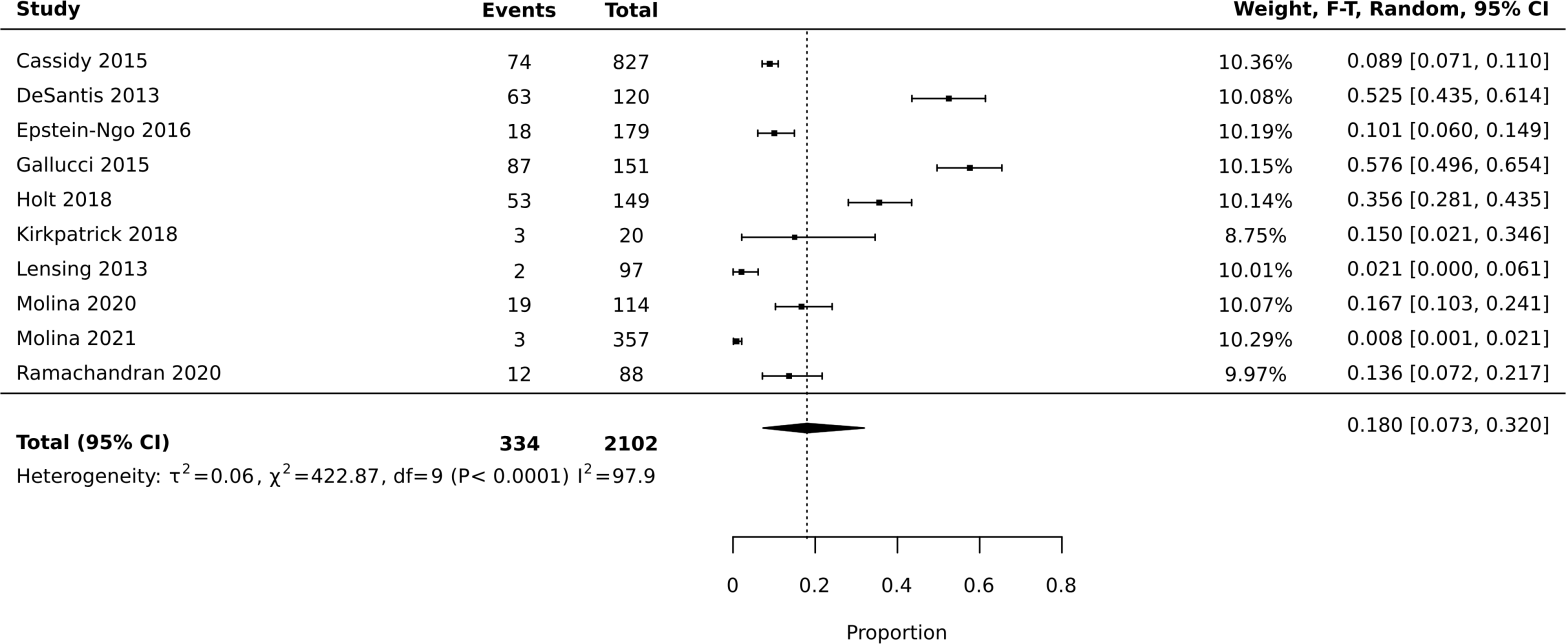
Meta-analysis of prevalence: past year and lifetime diversion.

**Figure 2:**
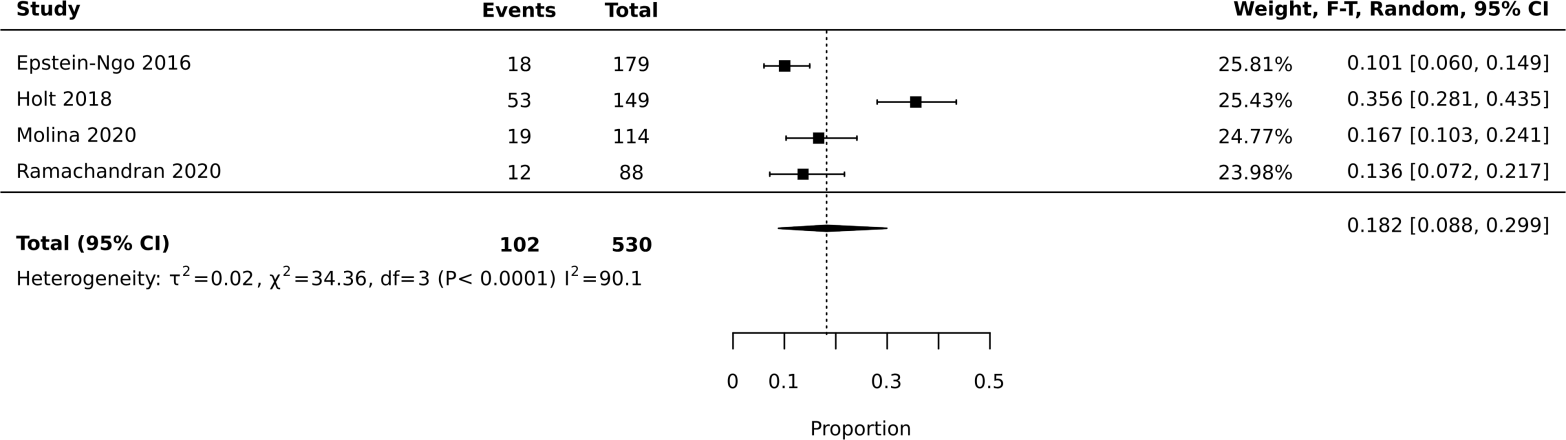
Meta-analysis of prevalence: past year diversion.

**Figure 3:**
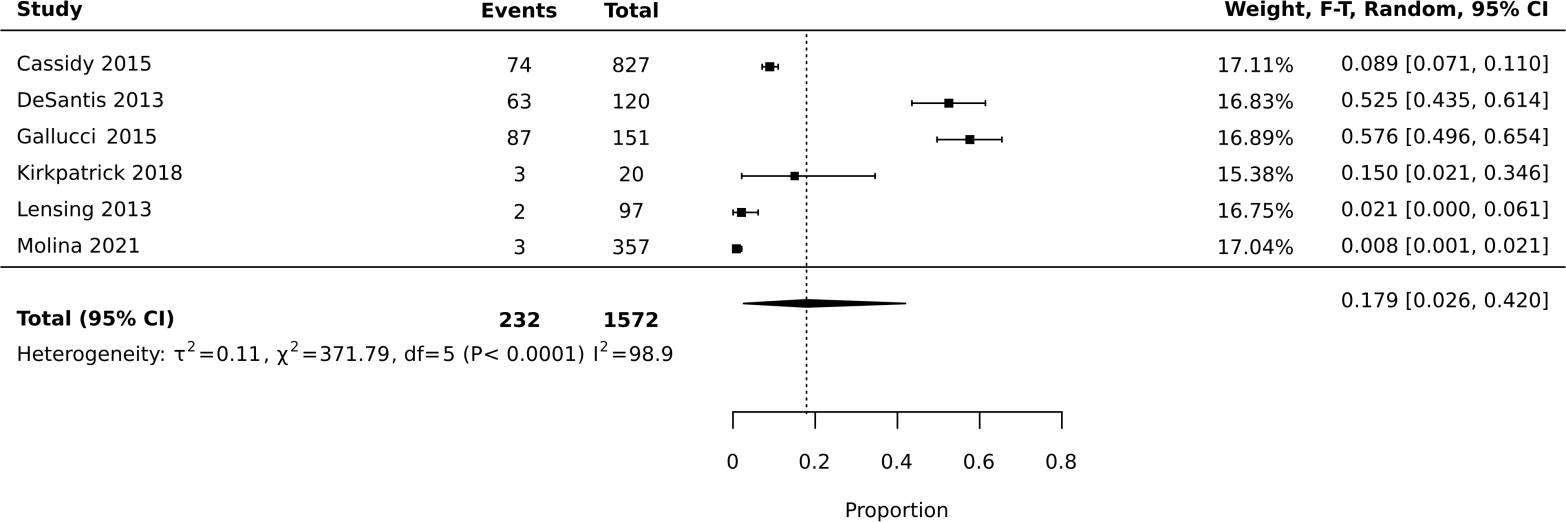
Meta-analysis of prevalence: lifetime diversion.

Meta-analysis of diversion studies was performed separately, with sensitivity analysis using different definitions of diversion (i.e. Lifetime vs past year – **Figures 1**–**3**). Funnel plot asymmetry for publication bias was not conducted due to the low number of included studies in the meta-analysis (28).

## 3 Results

### 3.1 Study selection

**Figure 4:**
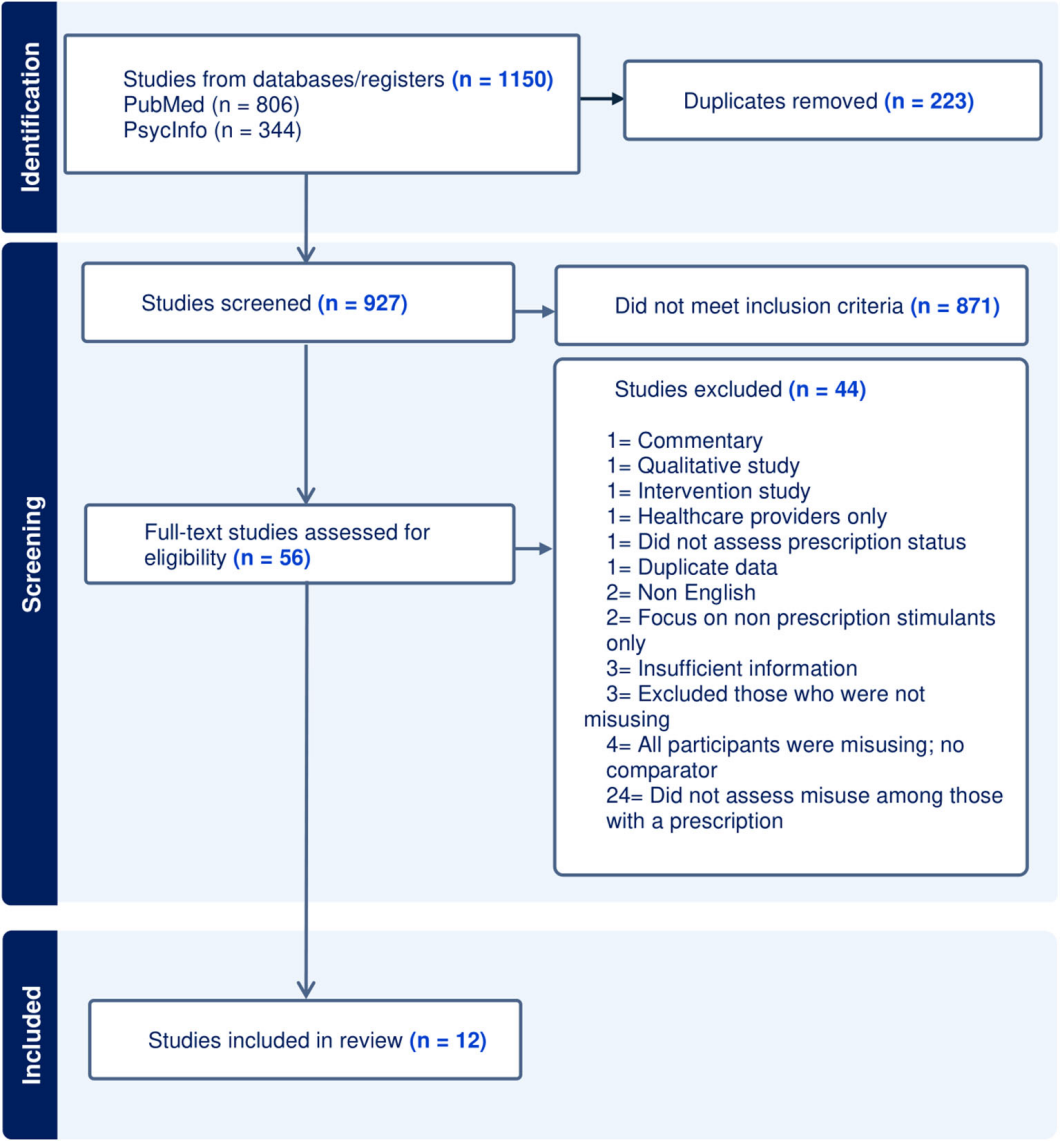
PRISMA flow diagram.

Abstracts and titles were screened for 927 citations (**Figure 4**). Reasons for exclusion at this stage (n = 871) were primarily due to an absence of prescription stimulant misuse or diversion information mentioned in the abstract or title. The most common reason for exclusion at full text stage (n = 24) was that misuse was not explored in the subgroup of the study who were prescribed stimulant medication. Studies frequently considered individuals who were prescribed stimulant medication to be ‘controls’ or appropriate users of stimulants. The second most common reason for exclusion (n = 4) was absence of comparison population for those who were misusing or diverting medication. This meant that prevalence rates of misuse or diversion were unable to be calculated.

### 3.2 Study characteristics

There were 12 studies included in the systematic review and meta-analysis (**Tables 1**–**3**). Whilst the search criteria allowed for several study designs, all eligible full-text studies were cross-sectional surveys. Eight studies were online surveys, one study was paper based (29) and two studies did not specify if the survey was electronic or paper based (30, 31). There was no uniform or validated questionnaire employed across studies. Survey content typically included items on demographics, ADHD diagnosis, stimulant prescription status, and misuse and/or diversion. The full survey instruments were not typically published, and no study reported use of standardized tools or methods to control for social desirability bias.

**Table 1:** Results of individual studies - studies assessing both misuse and diversion.

Reference/country	Study population	Sample size	Terminology	ADHD assessment	Prevalence of misuse	Prevalence of diversion
Cassidy et al. (16) United States	Adults 18–49 representative of the US population based on demographics. 53.4% female, 46.6% male	10 000 827 were prescribed stimulant medication currently or in the past	Non-medical use (NMU): use of a prescription stimulant without prescription, for reasons other than prescribed, for nonmedical purpose (e.g., to ‘get high’). Diversion: selling, trading, or giving away own medication.	Adult ADHD Self-Report Scale (43) 620 reported an ADHD diagnosis in the past 5 years	11.7% (n=97) of those prescribed stimulant medication reported NMU*^$^* in the past year Of the 620 who reported ADHD, 13.3% (n=83) reported stimulant NMU*^$^* in the past year	8.9% (n=74) of those prescribed stimulant medication reported diversion in their lifetime
Holt et al. (35) United States	Students 18 years or older prescribed stimulant medication for ADHD, from 2 universities 64% female, 35% male, 1% other^%^	149 adults prescribed stimulant medication at any point during college	Medical misuse: taking higher doses or for reasons other than prescribed. Diversion: selling or giving away own medication.	Self-report of ADHD diagnosis	32% (n=47) reported misuse in the past year	36% (n=53) reported diversion in the past year
Gallucci et al. (29) United States	Undergraduates aged 18–24 enrolled at a large public university. 62.9% female, 37.1% male	1022 151 were current stimulant prescription holders	Non-Medical Use of Prescription Stimulants (NMUPS): increasing the dosage or taking for reasons other than prescribed, or using someone else’s medication. Diversion: selling or giving away own medication.	Self-report of ADHD diagnosis 142 (94%) prescription holders reported an ADHD diagnosis	57.6% (n=87) of those prescribed a stimulant medication reported NMUPS in their lifetime	Of the 151 with a current prescription, 89 (58.9%) had diverted during their lifetime, 49 (32.4%) had diverted during the previous 30 days, and 116 (76.8%) reported being approached to divert
Kirkpatrick and Boyd (32) United States	Undergraduate nursing students. Age not reported. 94.8% female, 5.2% male	249 24 had received a prescription for stimulant medication in the past 12 months. 20 responded to the questions about misuse.	Medical misuse: taking more medication than prescribed, using medication to get high, using medication to increase other drug or alcohol effects. Diversion: selling, giving away, or loaning own medication.	Not assessed	15% (n=3) of those prescribed a stimulant medication reported stimulant misuse in the past year	15% (n=3) reported diversion in their lifetime^’^
Ramachandran et al. (33) United States	Undergraduates 18 and older attending a campus pharmacy to fill a prescription for stimulant medication. Mean age 21.8, 42% female, 58% male	88	Non-Medical Use of Prescription Stimulant (NMUPS): Using without a prescription, taking more of one’s own prescription stimulant than prescribed, or taking for reasons other than prescribed. Diversion: selling, trading, or giving away own medication.	Self-report of ADHD diagnosis 92% reported an ADHD diagnosis	33% (n=29) reported NMUPS in the past year 34% (n=25) of those with ADHD reported NMUPS in the past year	18% (n=12) reported diversion in the past year 59% were approached for drug diversion in the past year

NMUPS, Non-Medical Use of Prescription Stimulants.

**Table 2:** Results of individual studies - studies assessing misuse only.

Reference/country	Study population	Sample size	Terminology	ADHD assessment	Prevalence of misuse
Benson et al. (39) United States	Undergraduate students aged 18 or older at a large, public university. 76% female	936 adults 101 reported current prescription for stimulant medication	Misuse: Using too much medication, using more often than prescribed, snorting, mixing with other drugs, using medication that was not prescribed	Administered Current Symptoms Scale (44)	34% (n=34) of those prescribed stimulant medication reported misuse in the past year.
Hartung et al. (34) United States	Undergraduate students aged 18-25 (mean age 19.72 years) from 4 universities. 65.2% female	1,153 adults 171 reported current prescription for stimulant medication.	Medical Misuse: taking higher doses or more frequently than prescribed. Non-medical misuse: obtaining and using stimulant medication without a prescription	Administered Current Symptoms Scale (44)	14% (n=25) of those prescribed stimulant medication reported misuse in the past year.

**Table 3:** Results of individual studies - studies assessing diversion only.

Reference/country	Study population	Sample size	Terminology	ADHD assessment	Prevalence of diversion
DeSantis et al. (37) United States	Undergraduate students from a large university. Ages not specified. 30.8% female, 69.2% male	2,139 undergraduate students 120 reported currently taking a prescribed ADHD stimulant.	Distribution: selling or giving away own medication.	Self-report of ADHD diagnosis 189 (8.2%) reported an ADHD diagnosis	52.5% (n=63) gave away stimulant medication in their lifetime. 39.2% (n=47) had sold medication. 52.5% (n=63) both sold and gave away.
Epstein-Ngo et al. (38) United States	Adolescents attending 5 public schools. Ages not specified. 49.2% male 50.8% female	4965 3.6% (n=179) diagnosed with ADHD and prescribed stimulants in the past 12 months.	Diversion: selling, trading or giving away medication	Self-report of ADHD diagnosis	10% (n=18) had diverted medication in the past 12 months 20% were approached to divert medication in the past 12 months.
Lensing et al. (30) Austria	Primary care physicians and adults with ADHD Mean age 37.6 52.8% male 47.2% female	159 adults with ADHD and treating physicians 97 treated with stimulant medication	Diversion: ever sold ADHD medication.	DSM IV diagnosis confirmed with primary care physician	1.9% (n=2) reported diversion of stimulant medication.
Molina et al. (36) United States	Adolescents aged 13–18 participating in a randomized controlled trial of a stimulant diversion prevention workshop. Median age 15. 75% male	357 adolescents and one parent (85% mothers)	Diversion: sharing, trading or selling medication	ADHD diagnosis obtained through healthcare records	Diversion was rare (1%, n=3) 7% (n 25) reported being approached to divert
Molina et al. (31) United States	College students 18–25 years treated for ADHD with a stimulant and their primary care providers across 6 practices 68% attending universities; 24% attending community college	114 adults treated with a stimulant medication for ADHD	Diversion: selling, sharing, or trading prescribed stimulant medication	Diagnostic or billing code on health care record relating to ADHD	16.7% (n=19) reported diverting over the past year. 52.6% (n=60) were approached to divert (range =1 to 106 times)

These 12 studies varied in survey design, methodology and terminology. Terminology included ‘misuse’, ‘medical misuse’, ‘non-medical use’, ‘diversion’ and ‘distribution’. Some definitions referred solely to misuse of one’s own prescribed medication (the focus of this study), while others encompassed the use of nonprescribed stimulant medication. Due to this inconsistency in terminology across studies, **Tables 1**-**3** provide a detailed description of the terminology employed in each study.

Studies variously explored misuse and diversion over the past 30 days, past year, or lifetime. Stimulant prescription could refer to holding a current prescription, having been prescribed a stimulant in the past year, or prescribed a stimulant in one’s lifetime. The time period assessed in each study is outlined in **Tables 1**-**3**.

Study populations ranged from 88 respondents to 10,000 respondents. Eleven studies were from the United States (US), and one was from Austria. Eight studies sampled university or college students, three sampled patients of primary care practices, one surveyed school students, and one surveyed adults across the US. Ten studies sampled adults aged 18 and older. Two studies sampled undergraduate students but did not specify ages. Adolescents were sampled in two studies.

Eleven studies explored the presence of ADHD in some way: six asked participants if they had a diagnosis of ADHD, two assessed symptoms of ADHD using a self-report scale, and three confirmed a diagnosis of ADHD with the treating healthcare practitioner or medical records.

### 3.3 Risk of bias in studies

The Joanna Briggs Institute (JBI) critical appraisal tool was used (**Table 4**). Methodological shortcomings identified in the studies included inadequate sample size and lack of comprehensive assessment for ADHD. Few (17%) studies had an adequate sample size regarding the subgroup of interest, as the number of individuals prescribed stimulant medication tended to be low. Methods used to determine ADHD diagnosis were assessed in question 6: ‘Were valid methods used for the identification of the condition?’. JBI guidance states: ‘If the outcomes were assessed based on existing definitions or diagnostic criteria, then the answer to this question is likely to be yes. If the outcomes were assessed using observer reported, or self-reported scales, the risk of over- or under-reporting is increased, and objectivity is compromised.’ (26) Two studies used self-report scales only to explore ADHD symptomatology. Although the use of standardized self-report scales are valuable in assessing ADHD, particularly when used alongside thorough clinical observations, these were assessed as invalid methods in accordance with the guidance. One study did not explore ADHD diagnosis or symptoms in any way and therefore was marked as an invalid method of identifying the condition (32). Nine (75%) of the studies were considered to use valid methods, asking participants if they had a diagnosis of ADHD and/or confirming with health records or provider.

**Table 4:** JBI critical appraisal checklist.

Reference	Q1.	Q2.	Q3.	Q4.	Q5.	Q6.	Q7.	Q8.	Q9.
Cassidy et al. (16)									
Holt et al. (35)									
Gallucci et al. (29)									
Kirkpatrick and Boyd (32)									
Ramachandran et al. (33)									
Benson et al. (39)									
Hartung et al. (34)									
DeSantis et al. (37)									
Epstein-Ngo et al. (38)									
Lensing et al. (30)									
Molina et al. (36)									
Molina et al. (31)									
Percentage with Yes response	42%	58%	17%	75%	58%	75%	100%	67%	58%

Green = Yes, Red = No, Orange = Unclear.

### 3.4 Prevalence of misuse

Six studies reported the prevalence of misuse of stimulant medication over the past year, while one study reported lifetime misuse of stimulant medication. The total number of current or previous prescription stimulant holders who responded to questions assessing misuse was 1,507. Prevalence rates of misuse by those prescribed stimulant medication ranged from 11% to 33% in the past year. The one study assessing lifetime misuse reported a prevalence of 57.6% (29).

**Figure 5:**
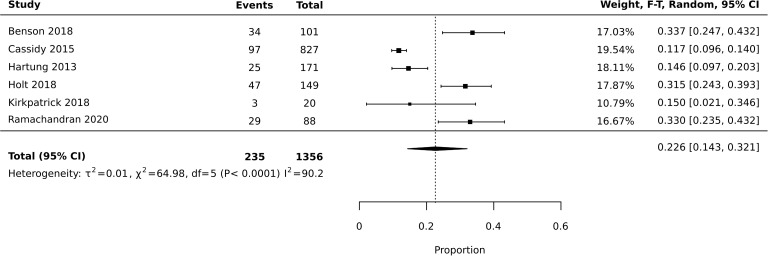
Meta-analysis of prevalence: past year misuse.

The six studies reporting past year prevalence were included for meta-analysis (**Figure 5**). Heterogeneity was high with I2 = 90.2 (X2 64.98). This may be explained by the differing terminology used to define misuse, demographics of participants, setting, and sample size. Random effects model was utilized in the context of high heterogeneity. Meta-analysis found an average prevalence of past year prescription stimulant medication misuse of 22.6%, with 95% confidence interval of 0.143-0.321 (**Figure 5**).

### 3.5 Prevalence of diversion

Six studies reported prevalence of diversion across the lifetime, and four reported prevalence of diversion in the past year. The total number of current or previous prescription stimulant holders who responded to questions assessing diversion was 1,813. Meta-analysis of all 10 studies reporting diversion (past year or lifetime) found an average estimated prevalence of 18%, with 95% confidence interval of 0.073-0.320 (**Figure 1**). Heterogeneity was high with I2 = 97.9 (X2 = 422.87). Meta-analysis of studies reporting past year diversion found an average prevalence of 18.2% with a 95% confidence interval of 0.088-0.299 (**Figure 2**). Meta-analysis of studies reporting lifetime diversion found an average estimated prevalence of 17.9% with confidence interval 0.026-0.420 (**Figure 3**).

### 3.6 Risk factors for misuse and diversion

Three studies explored risk factors for stimulant misuse in those with a diagnosis of ADHD and/or a current or previous prescription, and four studies explored risk factors for diversion. Risk factors for misuse included having an amphetamine-based stimulant prescription, comorbid depressive and anxiety symptoms, and beliefs that misuse was unlikely to be associated with risk of harm.

Ramachandran et al. surveyed students who were filling prescriptions for stimulants at a campus pharmacy. They found that 97% of those reporting misuse of their prescribed stimulant were filling a prescription for amphetamine-based stimulants, whilst only 3% were filling a prescription for methylphenidate (33). They found that those reporting misuse of their medication were more likely to report depressive and anxiety symptoms, and were more likely to believe that nonmedical use was associated with no or slight risk (41% vs 31%).

Hartung et al. assessed personality factors of perfectionism and ‘sensation-seeking’ and found no significant association with misuse (34). They also explored the contribution of ADHD symptoms with an 18-item self-report measure and found no significant association with misuse (30).

In terms of diversion, there were conflicting findings regarding the impact of gender, substance use, and misuse of medication. Three studies found no increased risk of diversion associated with gender (29, 35, 36). In contrast, one study of university students found male gender was significantly associated with increased risk of diversion (X2 = 13. 061, df = 1, p = 0.0001) (37). One study found that substance use including alcohol, marijuana, ecstasy and painkillers was significantly associated with diversion (37). Interestingly, students who used cocaine were less likely to divert medication. The authors hypothesized that these students may have been using their stimulants as a substitute for cocaine and therefore had less medication available for diversion.

Gallucci et al. found that individuals who reported misusing stimulant medication were five times more likely to divert (OR 4.967, CI 2.149–11.48, p < 0.001) than those with no misuse history. DeSantis et al. found that students who misused their own medication were 2.51 times (SD = 0.38) more likely to divert medication (37). In contrast, Holt et al. found no increased risk of diversion when individuals reported misuse of their own medication (35).

Having excess medication available, and exposure to peers who were misusing stimulant medication, emerged as risk factors for diversion. DeSantis et al. found that those who diverted medication were taking their stimulant less frequently than those who did not divert (4.7 days per week vs 5.3 days per week) (37). Holt et al. found that having others attempt to persuade the person to share or sell medication was associated with a higher risk of diversion (OR 1.888, CIs [1.132, 3.148]) (31). DeSantis et al. found that diverters reported that 50% (SD = 25.6) of their friends were using stimulant medication without a prescription, compared to only 26% (SD = 26.21) of friends of those who were not diverting (37).

Three studies reported that participants frequently experienced being approached to divert medication (requested to give away or sell). Gallucci et al. found that of 151 prescription holders, 94% of whom reported a diagnosis of ADHD, 58.9% had diverted during their lifetime, and 76.8% had been approached to divert (29). Ramachandran et al. surveyed 88 undergraduate students waiting to fill their prescription for stimulant medication at a university pharmacy, 92% of whom reported a diagnosis of ADHD. 18% reported diversion in the past year, while 59% were approached to divert (33). Epstein-Ngo et al. (38) sampled adolescents from 5 public schools, 179 of whom reported a diagnosis of ADHD for which they were prescribed stimulants in the past 12 months. 10% reported diverting their stimulant medication in the past year, while 20% reported being approached to divert. This suggests that rates of diversion are often much lower than rates at which individuals are being requested to share or sell medication.

## 4 Discussion

This is the first systematic review and meta-analysis of misuse and diversion of prescription stimulant medication among those with a current or previous prescription for stimulant medication for treatment of ADHD. Previous systematic reviews have found highly variable prevalence rates of diversion and misuse. In 2008, a systematic review (13) explored past year stimulant misuse in general populations and found prevalence of misuse ranged from 5 – 9% in school aged young people, and 5 – 35% in young adults. 11 – 29% of participants had sold their prescribed stimulant medication, and up to 23% of young people with prescriptions for stimulants for ADHD had been requested to sell, trade or give away medications. A 2012 systematic review (23) reported a 44% rate of misuse among those prescribed stimulant medication based on one adult study, and a rate of 3% from a study of young people aged 10-21. A 2020 review found that 4-35% of individuals reported non-medical use of their own prescription stimulant (14). Our meta-analysis of the available literature found an estimated prevalence of past year misuse of prescription stimulants to be 22.6% and an estimated prevalence of diversion to be 18.2%.

In terms of identified risk factors, this study adds to previous literature regarding risk factors for stimulant misuse and diversion. Substance use has been identified as a risk factor for stimulant misuse in the general population (13). This review found that the relationship between substance use and misuse or diversion of one’s own medication is unclear. Few studies explored the relationship between substance use and misuse or diversion of prescribed medication, and sample sizes were small. Findings from one study in this review suggested that while certain types of substance use may increase the risk of diversion, comorbid cocaine use decreased the risk (37). These results highlight the variability of the relationship between substance use and stimulant misuse and diversion. Three studies reported that individuals were approached to divert up to twice as often as they actually diverted (29, 33, 38). Individuals were more likely to divert if their peers were misusing stimulant medication (37). These findings suggest a potential role for supporting individuals to develop responses to requests to divert.

### 4.1 Limitations

There was a high degree of heterogeneity (I² > 90%) between the 12 studies included in meta-analysis. The high heterogeneity is attributable to variation in methodology, sample population, sample size, terminology and definitions. Sample size varied from 20 to 827. Definitions varied, and the differences in definitions and terminology are described in Column 4 of **Tables 1**-**3**. Survey questions differed across studies and were not routinely available as published data. Sampling strategies varied, and populations varied in terms of age and setting. The high heterogeneity warrants cautious interpretation of the pooled prevalence estimates, as the applicability of the findings may be limited in diverse contexts.

In terms of behaviors that carry the most risk, only two studies asked about route of administration of stimulant medication e.g. snorting, smoking or injection (16, 39) with one study reporting that up to 6% of individuals who reported misuse were injecting medication (16). The absence of inquiry about route of administration in the other surveys highlights the variability of the definitions utilized, and the difficulty in interpreting the level of risk accompanying misuse. Similarly, frequency of misuse and diversion was not explored in the majority of studies.

It is possible that studies that found higher rates of misuse and/or diversion were more likely to be published than those that found the risk was lower or negligible. This potential for publication bias could have contributed to an overestimation of the prevalence of stimulant medication misuse or diversion in our review. Although we conducted a comprehensive search of multiple databases to minimize the exclusion of unpublished studies, the small number of included studies precluded the use of a funnel plot to formally assess publication bias. Therefore, it is possible that publication bias impacted the results, contributing to higher estimates than would be found in comparable settings where publication is not a factor in distribution of results.

Studies rarely described how they supported participants to correctly identify ‘stimulant medication’. One study used photographs to aid the correct identification of stimulant medication (16), one study utilized health records to confirm prescription history (30), and the remainder either assessed prescription status by giving participants brand names of stimulant medications, or did not describe methods used to help participants correctly report stimulant medication history.

Few studies explored the exact stimulant that a participant was prescribed, or associated the stimulant with the participant’s response and risk of misuse or diversion. This limited the possibility of disaggregation of the results by stimulant. As a result, it was not possible to obtain meta-analysis results regarding which stimulants were associated with higher risk of misuse and/or diversion.

A notable limitation of the current body of literature, and consequently this review, is the limited geographical and demographic representation of the included studies. Most research has been conducted within the United States and predominantly involves college student populations. 11 out of 12 studies were US-based, with one study from Austria. Eight of the 12 studies focused on university/college students. The predominance of studies in college students has been noted in previous reviews (13, 23). This narrow focus restricts the generalizability of the findings to diverse populations and sociocultural environments.

There was considerable heterogeneity in how misuse and diversion were measured across studies. None of the included studies used validated or standardized tools to assess these behaviors, and most relied on author-developed questionnaires, the content of which was not always fully reported. Timeframes and definitions also varied, complicating cross-study comparisons. Furthermore, no study explicitly addressed the risk of social desirability bias, such as through anonymity assurances, social desirability scales, or indirect questioning methods. These methodological limitations may have contributed to underreporting of sensitive behaviors and may limit the generalizability and reliability of prevalence estimates”.

This study sought to examine misuse and diversion of stimulants amongst individuals of all ages. However, only two of the included studies explored misuse and diversion among children and adolescents. Limited information was available regarding the ages of participants in a number of studies. The data that was available regarding age of participants is described in column 2 of **Tables 1**-**3**. The small number of studies with children and adolescents, and the limited information about participants of older ages, limits the ability to generalize findings to individuals across the age spectrum.

Small sample sizes present an additional limitation. Although the overall sample size of the study may have been large, the subpopulation of interest tended to be relatively small, ranging from 20 to 827. These smaller sample sizes contributed to wide confidence intervals within the individual studies and the meta-analyses.

This study sought to focus on the specific population of individuals with a diagnosis of ADHD. However, methods used to explore or diagnose ADHD differed. Only three of the 12 studies utilized health records or confirmed the diagnosis with a clinician. Not all studies explored ADHD diagnosis in any form. Six studies relied on self-report of diagnosis. Two studies involved a self-report rating scale of ADHD symptoms, without explicitly addressing if the person had a diagnosis of ADHD. One study did not assess history of ADHD in prescription holders either through self-report or self-assessment. Self-reports could be influenced by recall inaccuracies or misunderstanding of diagnostic criteria, leading to possible misclassification. Assessment scales, whilst useful screening tools, cannot be substituted for a specialist diagnosis using international standards such as the DSM-5.

Within studies that did explore ADHD history, not all individuals with a prescription for stimulant medication reported a diagnosis of ADHD. In Australia and the US, stimulants may be approved for both ADHD and narcolepsy, and off-label prescribing may expand the potential indications. Narcolepsy is a relatively rare condition in comparison to ADHD, with estimated prevalence of 0.079% (40) versus estimated ADHD prevalence of 4-11% (41, 42). It is likely that very few individuals in the included studies were prescribed stimulant medication for reasons other than ADHD, however, this remains a possibility and may have influenced the findings.

The variability in methods used to explore ADHD, and the presence of participants who were prescribed stimulant medication but did not report a history of ADHD, may affect the internal validity of the findings.

### 4.2 Implications for practice

This study highlights the importance of risk assessment for misuse and diversion prior to prescription of stimulant medication for individuals with ADHD. Possible risk factors identified in this review were substance use, peer misuse of stimulants, personal beliefs about the risks of misuse and diversion, having excess medication at home due to missed doses, and past history of misuse of stimulant medication.

Notably, there is evidence that the risk assessment process itself has the potential to mitigate the risk of misuse and diversion. One study found that individuals were less likely to divert medication if their doctor frequently asked if they ever ‘share’ their medication with others, and if they understood the dangers associated with sharing stimulants with nonprescribed individuals (37). This aligns with findings that when healthcare providers delivered brief interventions aimed at reducing diversion risk, patients were less likely to report an intention to divert (31).

Comorbid substance use is currently recognized as a risk factor for misuse and diversion of stimulant within existing guidelines (5, 6). Considering the findings in this review, risk assessment should also include exploration of an individual’s history of stimulant misuse, peer misuse, and beliefs about potential consequences of misuse and diversion of stimulants. When enquiring about adherence with treatment, prescribers might also ask about leftover medication or medication ‘stockpiles’, which may increase the risk of the individual giving away or selling medication. Education of individuals about the risk of serious adverse outcomes should oral medication be administered intravenously, either by the individual or a recipient of diverted medication, is recommended. Regular assessment and open communication between patients and healthcare providers can play a pivotal role in reducing these risks, acting as a brief intervention, as well as risk assessment. These practical recommendations are echoed by the studies included in this review. Further elaboration of these suggestions is highlighted in several studies (31, 34, 35, 37). A standardized checklist could be useful as an opportunity for brief intervention at clinical encounters. We propose a brief intervention that utilizes the pneumonic ‘FOCUS’ (**Box 1**).

Box 1 **FOCUS standardized checklist.**

F – Frequency of use

How often and at what doses is the patient using medication across a month. Are doses skipped or excess stockpiles of medication available at home, or are scripts required at shorter intervals than expected.

O- Off label use

Does the patient use their stimulant medication to stay awake, increase energy, or for other purposes not directly related to ADHD treatment?

C- Coercion

Has the patient experienced pressure for others to share their medication?

U- Understanding of risks

Explore the patient’s understanding of the risks of misuse and diversion, and provide education. In particular, the risk of either the person, or a someone who receives the medication as a result of diversion, using the medication in a route not recommended, such as intravenous, which can be fatal.

S – Safety planning

Discuss strategies to support the person to address potential risk of misuse or diversion. For example, reducing the number of tablets prescribed, storing medications securely so that there is reduced opportunity for others to request medication, taking extended release formulations so that short acting medications do not need to be taking at school or education settings where peer pressure may be a concern.

### 4.3 Future directions

This review found that few studies have explored risk factors for misuse and diversion within the population of those who are prescribed stimulant medication for a diagnosis of ADHD. There is a paucity of data relating to individuals prescribed stimulant medication for ADHD who are not in higher education. There is also limited data relating to children and adolescents with ADHD who are prescribed stimulants. Where young children and adolescents are prescribed stimulant medication, their parent or carer is likely to be managing stimulant medication, and likely to be the individual most at risk of diversion and misuse. Further studies involving children and parents would strengthen the current knowledge base around risks in this demographic.

Further research that utilizes standardized clinical evaluations to identify individuals with a diagnosis of ADHD would strengthen the reliability of conclusions drawn. Future research should prioritize including a wider variety of populations across different age ranges and cultural backgrounds. Research in diverse geographic and demographic populations will inform a more nuanced and culturally informed risk assessment for misuse and diversion of stimulant medication.

Future research would be strengthened by a more uniform definition of key terms such as misuse and diversion. We recommend that future studies use the terminology adopted in this review to promote consistency across research. Misuse is defined as the use of prescribed stimulants in a manner not intended by the prescriber. This includes consumption in excess of the prescribed dose, administration via non-oral routes (e.g., intranasal or injection), use for purposes other than the treatment of ADHD, or concurrent use with alcohol or other substances. Diversion is defined as the transfer of stimulant medication prescribed for ADHD from the individual for whom it was prescribed to another individual without a prescription, typically through giving away or selling the medication. These definitions encompass the full range of potentially problematic or concerning behaviors associated with the use of stimulant medications outside the parameters of medical guidance. Study designs that explicitly differentiate between various forms of misuse and diversion would enhance the clinical applicability of the findings. Studies that specifically explore intravenous use of stimulants, which is associated with substantial morbidity and mortality, would address an important and under-studied area of concern.

## 5 Conclusion

This systematic review sheds light on a critical and understudied issue – the misuse and diversion of prescription stimulant medication among individuals with a current or previous prescription for the treatment of ADHD. The adverse effects of misuse and diversion pose significant health risks. The findings of this review suggest that risk assessment should consider factors such as comorbid substance use, peer influence, personal beliefs around potential consequences of misuse and diversion, and previous experiences of misusing stimulant medication. Further research in diverse populations and age groups would provide a more comprehensive understanding of this issue. Improved understanding of risk factors for misuse and diversion in those prescribed stimulant medication for ADHD would support the development of risk assessment tools and targeted interventions.
